# Increased levels of PD1 and glycolysis in CD4^+^ T cells are positively associated with lymph node metastasis in OSCC

**DOI:** 10.1186/s12903-023-03043-6

**Published:** 2023-06-03

**Authors:** Kun Wu, Nannan Han, Yuanyuan Mao, Yan Li

**Affiliations:** 1 Department of Oral and Maxillofacial Surgery, Second Xiangya Hospital of Central South University, Changsha, China; 2 Department of Oral and Maxillofacial-Head and Neck Oncology, Shanghai Ninth People’s Hospital, School of Medicine, Shanghai Jiao Tong University, Shanghai, China; 3 Department of Anesthesiology, Second Xiangya Hospital of Central South University, Renmin road, No. 139, Changsha, Hunan 410011 China; 4 Precision Research Center for Refractory Diseases, Shanghai General Hospital, Shanghai Jiao Tong University School of Medicine, Shanghai, China

**Keywords:** oral squamous cell carcinoma, lymph node metastasis, CD4^+^ T cells, immune checkpoint, glycolysis

## Abstract

**Background:**

Cervical lymph node metastasis is one of the poorest prognostic factors in oral squamous cell carcinoma (OSCC). Activated immune cells generally have metabolic abnormalities in the tumour microenvironment. However, it is unknown whether abnormal glycolysis in T cells could facilitate metastatic lymph nodes in OSCC patients. The aim of this study was to investigate the effects of immune checkpoints in metastatic lymph nodes and determine the correlation between glycolysis and immune checkpoint expression in CD4^+^ T cells.

**Methods:**

Flow cytometry and immunofluorescence staining were used to analyse the differences in CD4^+^ PD1^+^ T cells between metastatic lymph nodes (LN^+^) and negative lymph nodes (LN^−^). RT‒PCR was performed to detail the expression of immune checkpoints and glycolysis-related enzymes in LN^+^ and LN^−^.

**Results:**

The frequency of CD4^+^ T cells decreased in LN^+^ patients (*p* = 0.0019). The PD1 expression of LN^+^ increased markedly compared to that of LN^−^ (*p* = 0.0205). Similarly, the PD1 of CD4^+^ T cells in LN^+^ increased significantly compared to that of LN^−^. Additionally, glycolysis-related enzyme levels in CD4^+^ T cells from LN^+^ patients were dramatically higher than those in LN^−^ patients. PD1 and Hk2 expression in CD4^+^ T cells was also increased in LN^+^ OSCC patients with prior surgical treatment compared to those without.

**Conclusions:**

These findings suggest that lymph node metastasis and recurrence in OSCC are associated with increases in PD1 and glycolysis in CD4^+^ T cells; this response may serve as a potential regulator of OSCC progression.

**Supplementary Information:**

The online version contains supplementary material available at 10.1186/s12903-023-03043-6.

## Introduction

Oral squamous cell carcinoma (OSCC) is a major and devastating oral cancer subtype, accounting for over 90% of all malignant tumours in the oral cavity [1]. Cervical lymph node metastasis is one of the poorest prognostic factors in OSCC, with a 50% reduction in the survival of patients with a lymph node-positive diagnosis compared to those without [2]. Accordingly, we have detailed the underlying molecular mechanism of cervical lymph node metastasis in an attempt to decrease the mortality of patients with OSCC.

Inflammation is one of the most notable characteristics of oral disease. Recent studies have shown the role of periodontitis and its mediators [3] (miRNAs, circulating cells) in gingival inflammation [4] and using the immunosuppressive drug, Tacrolimus, as a treatment for periodontitis and oral diseases that could be related to periodontitis and OSCC [5]. Host immune tolerance and activation depend on the balance of positive and negative signals, which are determined by immune checkpoints [6]. Malignant tumour cells evade antitumour immune responses by facilitating negative signals such as PD1/PDL1 [7]. More specifically, upregulation of PD1 inhibits the effector functions of T cells and expansion in the tumour microenvironment, thereby enabling tumour cells to escape immune surveillance [8]. Moreover, immune checkpoint receptors in T cells can determine their activation, expansion, and effector functions [9] by regulating metabolic activity [10, 11]. T cells have a highly dynamic metabolism and specific metabolic pathways that can support specific functions in various cells, such as effector, memory, regulatory, and alloreactive T cells [12]. Therefore, activating T cells causes a large increase in glucose metabolism and aerobic glycolysis [13]. Lymph nodes are pivotal peripheral immune organs that respond to disseminated tumour cells by presenting tumour cell antigens and subsequently prime effector cells, such as antigen-specific T cells [14, 15]. However, it remains unclear how immune checkpoints contribute to metastatic lymph nodes in patients with OSCC. Furthermore, the correlation between glycolysis and immune checkpoint expression in CD4^+^ T cells has not been explored. The aim of this study was to investigate the effects of immune checkpoints in metastatic lymph nodes and to determine this correlation.

## Materials and methods

### Ethics

The Ethics Committee of the Second Xiangya Hospital approved this study. Written informed consent was obtained from all participants prior to their enrollment. All experimental procedures were performed in accordance with the Helsinki Declaration.

### Patients and specimens

All OSCC samples were collected from the Department of Oral and Maxillofacial Surgery at Second Xiangya Hospital of Central South University. Specifically, lymph nodes were obtained from patients with OSCC who underwent surgery between June 2018 and July 2019. A single metastatic lymph node (LN+) and one paired negative lymph node (LN) were collected from 11 patients with OSCC. Half of each lymph node was stored for the experiments, and the other half was sent for pathological diagnosis. Clinical parameters were obtained from medical records. All these patients underwent neck dissection were included. The patients who had preoperative radiotherapy or chemotherapy were excluded.

### T cell isolation

Lymph nodes preparation and digestion were using 1 mg/ml Collagenase IV and 40 µg/ml Dnase I as followed [16]. The cells processed from the lymph nodes were used for T cell isolation and flow cytometry analysis. CD4^+^ T cells were sorted from a single-cell suspension drawn from lymph nodes with the CD4^+^ T cell Isolation Kit (BioLegend), purity levels were greater than 95%, as determined by using the BD FACSCalibur. CD4^+^ T cells were used for real-time PCR analyse.

### RNA extraction and real-time PCR (RT-PCR) analysis

Total RNA from CD4^+^ T cells was isolated using the TRIzol reagent (Takara, Japan), and cDNA was synthesized using a PrimeScript RT Reagent Kit (Takara, Japan). Real-time PCR was performed using a SYBR Premix Ex Taq Reagent Kit (Takara, Japan) via the StepOne Real-Time PCR System (Life Technologies, USA) according to the manufacturer’s instructions. In tissue lysates, mRNA levels were normalized to β-actin levels. The primer sequences used in this study are listed in Supplementary Table 1.

### Immunofluorescence analysis

Paraffin-embedded sections were deparaffinized, rehydrated, and submerged in an EDTA buffer for heat-induced antigen retrieval. The sections were then immersed in 0.3% hydrogen peroxide, blocked with 10% goat serum, incubated with specific primary antibodies at 4 °C overnight, and incubated with an Alexa Fluor 488-cojugated secondary antibody (Invitrogen, USA) or Alexa Fluor 549-cojugated secondary antibody (Invitrogen, USA) in the dark at room temperature. Sections were stained with DAPI (Sangon Biotech, China) to detect the nuclei. Sections were imaged using a TCS SP2 laser-scanning confocal microscope (Leica Microsystems, Germany) and Gen5 software (Bio Tek, USA).

### Flow cytometry

Cell surface markers were analyzed using flow cytometry (FCM). The living cells were stained with antibodies in PBS containing 0.1% (w/v) BSA and 0.1% NaN_3_ in 50µL FACS buffer for 30 min on ice. 7-Amino-Actinomycin D (7-AAD) was used for the exclusion of nonviable cells in flow cytometric assays. The following antibody-fluorochrome combinations were used: anti-CD4 BB515 (RPA-T4), anti-CD8a BB700 (RPA-T8), anti-CD19 Percp (HIB19), anti-CD20 FITC (2H7), anti-CD11c PE (3.9), anti-MHCII Percp (G46-6), anti-CD68 FITC (Y1/82A), anti-CD86 Percp (FUN-1), anti-CD274 FITC (MIH1), anti-CD279 APC (EH12.2H7), and anti-CD152 APC (BNI3). The antibodies were obtained from BioLegend or BD Pharmingen).

### Statistical analysis

Kruskal-Wallis, Mann-Whitney, or nonparametric paired tests (i.e., the Wilcoxon matched paired test) were used to analyze the non-parametric distribution of the samples. All statistical analyses were performed using SPSS (version 17.0; SPSS, Chicago, IL, USA). All values were two-sided, and statistical significance was set at *p* < 0.05.

## Results

### Metastatic lymph nodes in OSCC patients

**Fig. 1:**
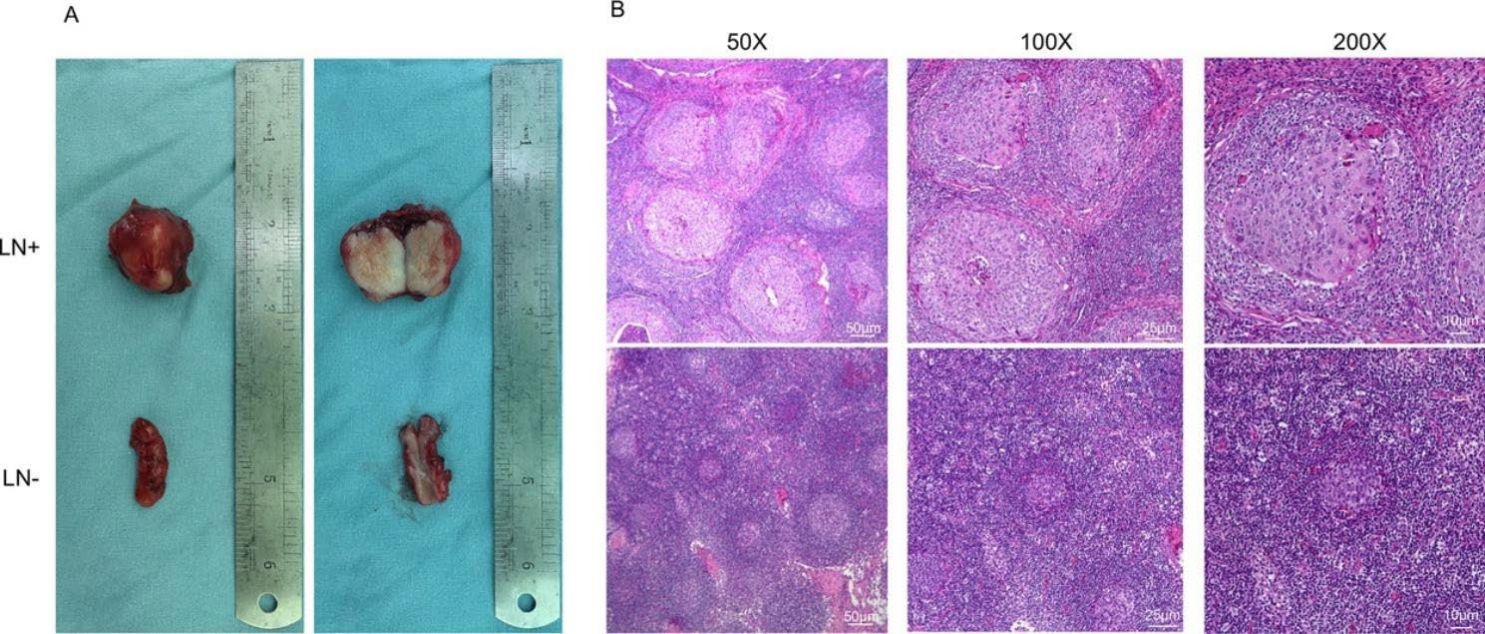
Metastatic lymph nodes (LN+) and negative lymph nodes (LN-) of OSCC patients. **(A)** Macroscopic view images of LN + and LN- fresh tissue samples from OSCC patients. **(B)** Representative hematoxylin & eosin staining (H&E) of LN + and LN- samples. n = 11 (Resolution: 300 dpi)

Lymph node metastasis is one of the poorest prognostic factors in patients with OSCC [2]. Fresh samples of metastatic lymph nodes (LN^+^) and negative lymph nodes (LN^−^) were collected, and keratinizing cells were observed in LN^+^ (Fig. 1A and B). The overall size of LN^+^ samples were larger than that of LN^−^ samples. The section slices of LN^+^ were observed to be rougher and harder than that of LN^−^. The presence of cancer nests were also found in LN^+^ samples, while lymphoid follicles were shown in LN^−^.

### Decreased frequency of CD4^+^ T cells in metastatic lymph nodes

**Fig. 2:**
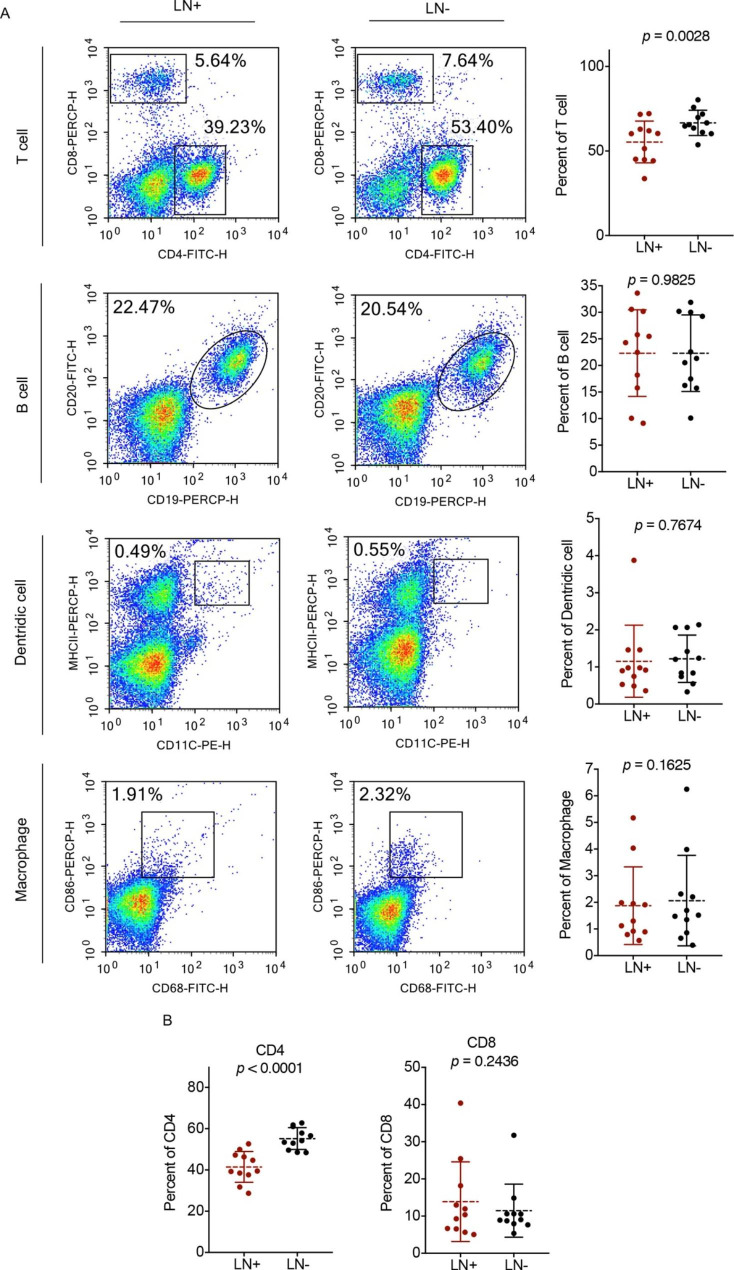
Immune cell types of LN + and LN- from OSCC patients. **(A)** Cells of LN + and LN- from OSCC patients stained with 7AAD, anti-CD45 mAb, anti-CD4 mAb, anti-CD8 mAb, anti-CD19 mAb, anti-CD20 mAb, anti-CD11c mAb, anti-MHCII mAb, anti-CD68 mAb and anti-CD86 for flow cytometry analysis. Representative flow cytometry analyse of T cells, B cells, Dentridic cells, and macrophages isolated from LN + and LN- in OSCC patients. **(B)** Percentages of CD4 and CD8 cells analyzed in LN + and LN- samples. n = 11. The data present as mean ± SD, the data were analyzed by t-test

The lymph node is a secondary lymphoid organ [17] that represents a pivotal meeting point of various immune cell types for adaptive immune responses [18]. To identify the immune cell types in metastatic lymph nodes, LN^+^ and paired LN^−^ collected from each OSCC patient were analysed via flow cytometry. The clinical parameters of the 11 OSCC patients are shown in Table 1. The percentage of T cells was significantly lower in LN^+^ than in LN^−^ (*p* = 0.0028) (Fig. 2A). However, there was no significant difference between the percentage of B cells (*p* = 0.9825), dendritic cells (*p* = 0.7674), or macrophages (*p* = 0.1625) between LN^+^ and paired LN^−^ (Fig. 2A). In a further analysis, CD4^+^ T cells dramatically decreased in LN^+^ compared with LN^−^ (*p* < 0.0001) (Fig. 2B), whereas the presence of CD8^+^ T cells did not change in LN^+^ (Fig. 2B). This result indicated that the decreased frequency of CD4^+^ T cells was closely associated with metastatic lymph nodes in OSCC.

**Table 1:** The clinical parameter of OSCC patients were used

NO.	Age	Gender	T classification	Lymph node status	Tumor site	Perineural invasion	Prior Radiotherapy/ Chemosensitivety	Prior surgical treatment
1	78	Female	T2	pN positive	Tongue	No	No	Yes
2	49	Female	T3	pN positive	Buccal mucosa	Yes	No	Yes
3	51	Male	T3	pN positive	Tongue	Yes	No	Yes
4	59	Male	T3	pN positive	Tongue	Yes	No	Yes
5	71	Male	T2	pN positive	Tongue	Yes	No	Yes
6	68	Male	T2	pN positive	Tongue	Yes	No	No
7	85	Male	T4	pN positive	Buccal mucosa	No	No	Yes
8	45	Female	T3	pN positive	Oral floor	No	No	No
9	52	Male	T2	pN positive	Tongue	No	No	No
10	66	Male	T4	pN positive	Tongue	No	No	Yes
11	58	Female	T3	pN positive	Gingiva	No	No	No

pN: pathology lymph nodes status

### Increased frequency of PD1 in CD4^+^ T cells occurred in metastatic lymph nodes

**Fig. 3:**
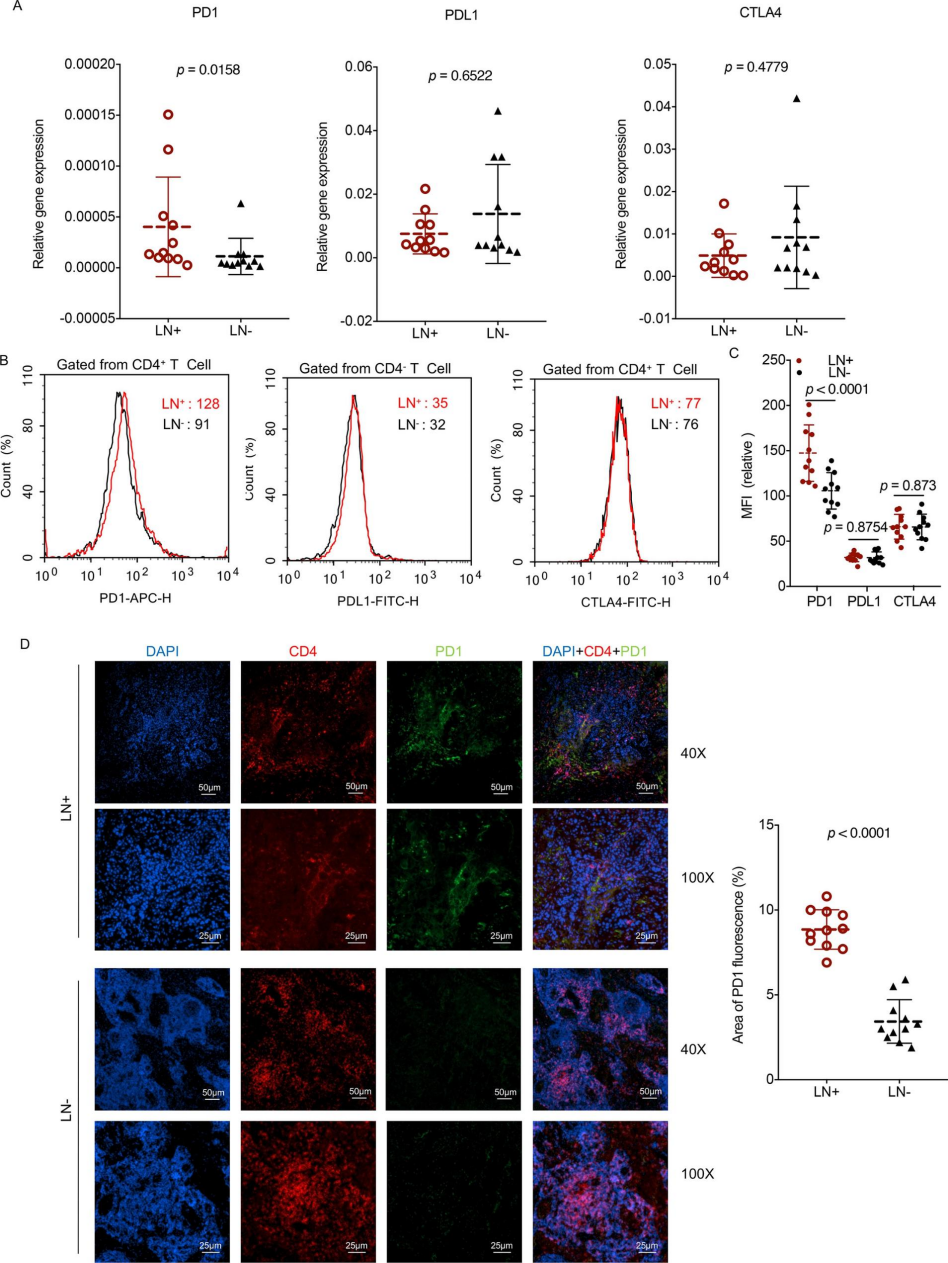
Immune checkpoint expression of LN + and LN- from OSCC patients. **(A)** mRNA expression of PD1, PDL1 and CTLA4 as performed by RT-PCR. **(B)** PD1, PDL1 and CTLA4 of LN + and LN- measured through flow cytometric analysis. **(C)** PD1and CD4 of LN + and LN- detected using immunofluorescence analysis. n = 11, (Resolution: 300 dpi). The data present as mean ± SD, the data were analyzed by t-test

Immune checkpoint receptors on T cells can negatively determine their expansion, activation, and effector functions via inhibitory signals generated through binding to their receptors [9]. The interaction of PDL1 with its cognate ligand PD1 on activated T cells inhibits antitumour immunity by counteracting T-cell-activating signals [19]. To detail the expression of the immune checkpoint receptors PD1, PDL1, and CTLA4 in LN^+^, immune checkpoint receptor transcriptional levels were detected using RT‒PCR. The LN^+^ and paired LN^−^ from each OSCC patient were collected, and the clinical parameters of 11 OSCC patients are shown in Table 1. Only the PD1 expression level of CD4^+^ T cells was notably upregulated in LN^+^ compared to LN^−^ (*p* = 0.0158) (Fig. 3A). To further determine changes in immune checkpoints in metastatic lymph nodes, PD1, PDL1, and CTLA4 protein levels were detected using flow cytometry. As expected, the PD1 protein level of CD4^+^ T cells was significantly upregulated in LN^+^ compared to LN^−^ (*p* < 0.0001) (Fig. 3B and C, respectively). However, there was no significant difference in PDL1 and CTLA4 between LN^+^ and LN^−^ (Fig. 3B and C, respectively). Immunofluorescence analysis showed that PD1 was predominantly expressed in CD4^+^ T cells and markedly upregulated in LN^+^ cells compared to LN^−^ cells (Fig. 3D). Our findings revealed that the increased PD1 of CD4^+^ T cells in LN^+^ was related to lymph node metastasis progression.

### Elevated glycolysis-related enzyme levels in CD4^+^ T cells from metastatic lymph nodes

**Fig. 4:**
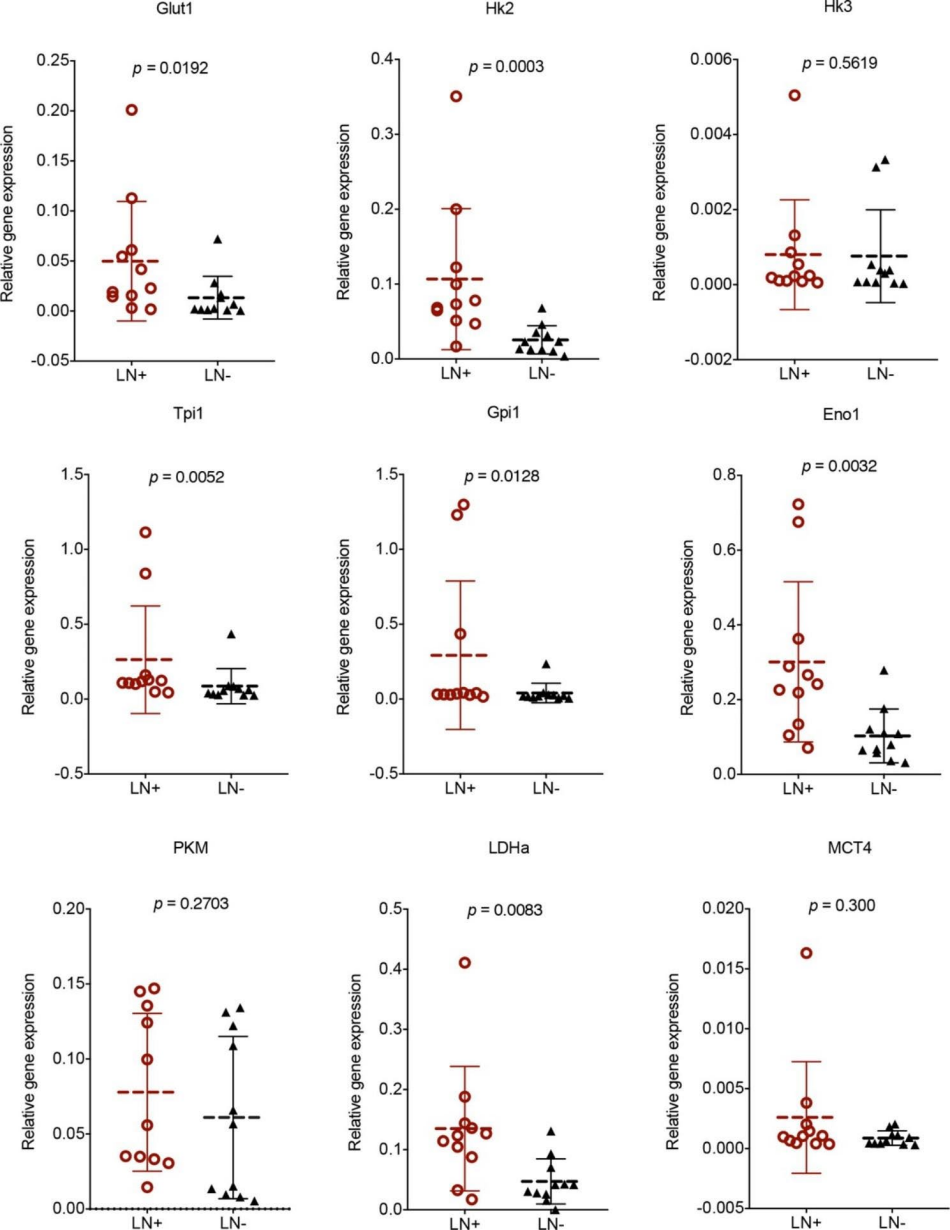
The mRNA expression of Glut1, Hk2, Hk3, Tpi1, Gpi1, Eno1, PKM, LDHa and MCT4 in CD4^+^ T cells as detected by RT-PCR. n = 11. The data present as mean ± SD, the data were analyzed by t-test

T cells depend on dramatic increases in glucose metabolism as fuel to support the growth, function, survival, and differentiation of activated T cells [12, 20]. To determine whether glycolysis-related enzymes contribute to CD4^+^ T cells in metastatic lymph nodes, the mRNA expression levels of Glut1, Hk2, Hk3, Tpi1, Gpi1, Eno1, PKM, LDHa, and MCT4 in CD4^+^ T cells were detected via RT‒PCR. The mRNA expression levels of Glut1, Hk2, Tpi1, Gpi1, Eno1, and LDHa in CD4^+^ T cells dramatically increased in LN^+^ compared to LN^−^ (Fig. 4). While there was no significant difference between Hk3, PKM, and MCT4 expression levels in LN^+^ and LN^−^, the average Hk3, PKM, and MCT4 expression levels in LN^+^ were higher than those in LN^−^ (Fig. 4). These results suggest that an increase in PD1 in CD4^+^ T cells is linked to glucose metabolism and aerobic glycolysis.

### PD1 and Hk2 expression of CD4^+^ T cells in metastatic lymph nodes of OSCC patients with prior surgical treatments compared to those without

**Fig. 5:**
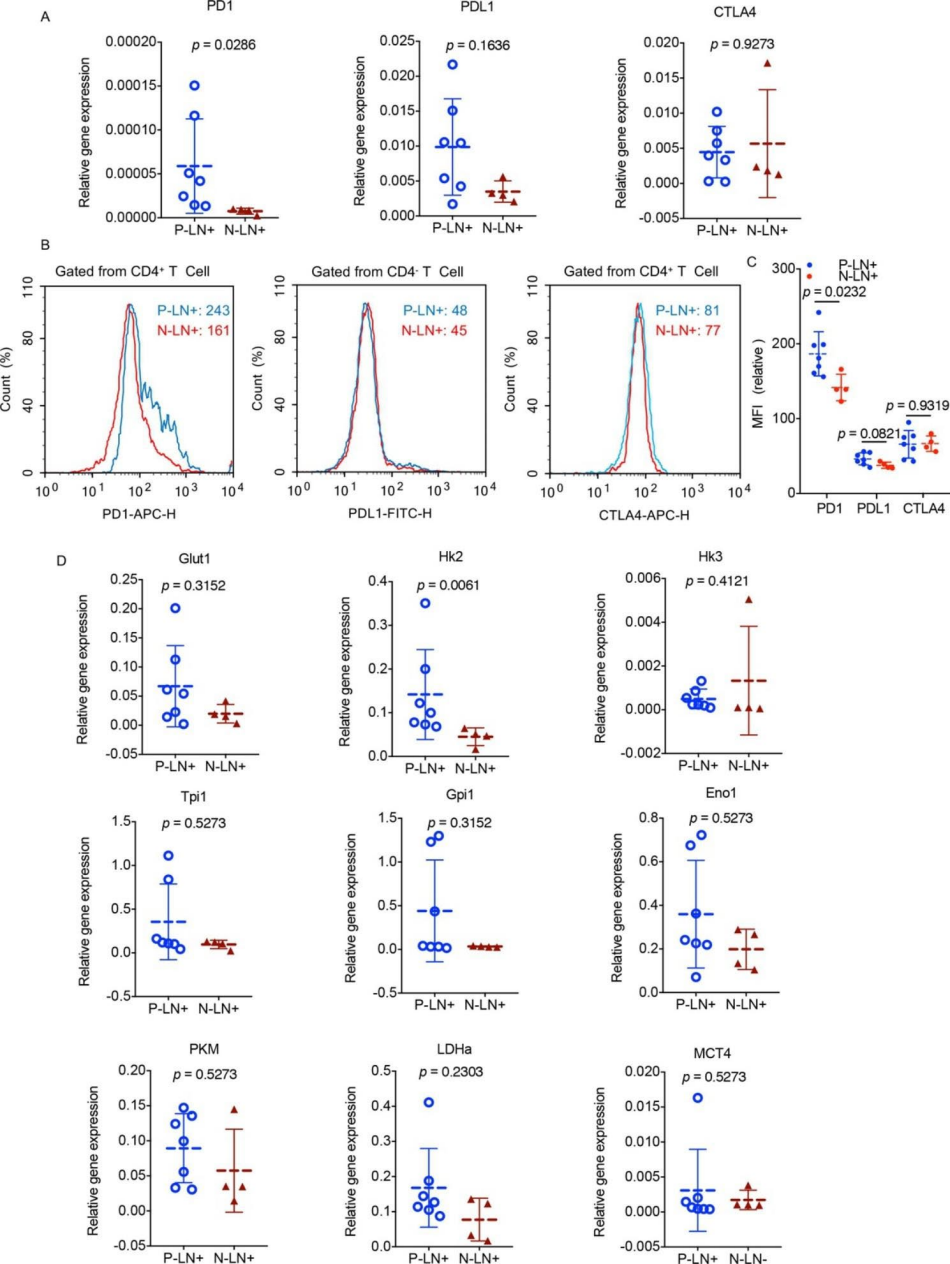
Immune checkpoint and glycolysis related enzymes analyzed according to prior surgical treatment history. **(A)** mRNA expressions of PD1, PDL1 and CTLA4 in LN + and LN- as performed by RT-PCR according to the surgical treatment history. n = 11. **(B)** PD1, PDL1 and CTLA4 of LN + and LN- as measured via flow cytometric analysis according to the surgical treatment history. n = 11. **(C)** Glut1, Hk2, Hk3, Tpi1, Gpi1, Eno1, PKM, LDHa and MCT4 of CD4^+^ T cells in LN + and LN- as detected by RT-PCR according to the surgical treatment history. n = 11. The data present as mean ± SD, the data were analyzed by t-test

LN^+^ in OSCC patients with a surgical treatment history (i.e., underwent neck lymph node dissection) was defined as P-LN^+^ (n = 7), and LN^+^ in OSCC patients without a prior surgical treatment history was defined as N-LN^+^ (n = 4). The PD1 expression level of CD4^+^ T cells was markedly upregulated in P-LN^+^ compared to N-LN^+^ (*p* = 0.0286), whereas no significant difference in PDL1 and CTLA4 expression between P-LN^+^ and N-LN^+^ was found (Fig. 5A and B C, respectively). To determine whether glycolysis-related enzymes contributed to the upregulation of PD1 in CD4^+^ T cells in P-LN^+^, the mRNA expression levels of Glut1, Hk2, Hk3, Tpi1, Gpi1, Eno1, PKM, LDHa, and MCT4 in CD4^+^ T cells were analysed according to the patients’ surgical treatment history. Only Hk2 expression levels of CD4^+^ T cells were found to dramatically increase in P-LN^+^ compared to N-LN^+^ (*p* = 0.0061) (Fig. 5D). These data suggest that the increase in PD1 in CD4^+^ T cells in P-LN^+^ was associated with elevated Hk2.

## Discussion

We have demonstrated in this study that the percentage of CD4^+^ T cells decreased in LN^+^ compared to LN^−^. Additionally, the expression of PD1 and glycolysis-related enzymes was elevated in CD4^+^ T cells from metastatic lymph nodes. These results indicate that increases in PD1 in CD4^+^ T cells in LN^+^ facilitate lymph node metastasis progression and may be correlated with the glycolysis level. In the performed experiments, the findings revealed that PD1 and Hk2 in CD4^+^ T cells were upregulated in P-LN^+^ compared to N-LN^+^ cells. These results indicated that lymph node metastasis and recurrence in OSCC are associated with increases in PD1 and glycolysis in CD4^+^ T cells.

Inflammation is a fundamental characteristic of oral disease. Oral disease-relevant biomarkers, such as miRNAs, circulating cells, and Tacrolimus as an anti-inflammatory agent, could improve the early diagnosis and treatment of oral diseases [3–5]. T cells are regarded as the principal weapons of immunity against cancer [21]. T cells inhibit tumour cells in various ways, either directly by killing tumour cells via cytolytic mechanisms or indirectly by modulating the tumour microenvironment [22]. Emerging evidence has revealed that CD4^+^ T cells are necessary to initiate and maintain anticancer immune responses [23, 24]. Changes in the number of CD4^+^ T cells are vital in creating robust hosts against tumours, especially for lymph node metastasis [25]. Our findings suggest that a decrease in the percentage of CD4^+^ T cells in LN^+^ inhibits antitumour immunity, which leads to the progression of OSCC. Tumour-draining lymph nodes are the major sites for priming tumour-reactive T cells and tumour metastasis. LN^+^ contain tumour cells and immune cells, and the percentage of immune cells decreases correspondingly as tumour cells increase [26]. It is uncertain why the immune cells in the lymph nodes cannot recognize and clear the invading tumour cells and progress towards tumour metastasis.

PD-1 checkpoint blockades have revolutionized the field of cancer immunotherapy. As immune checkpoint blockade therapies fail to induce responses in the majority of cancer patients, increasing the objective response rate has therefore become an urgent challenge [27]. A previous study on cervical carcinomas reported that PD1 was expressed by a vast number of infiltrating CD8^+^ T cells, thus suggesting that PD1 could serve as a potential therapeutic target [28]. In addition, a recent study suggested that CD4^+^ T cells, as pivotal regulators of PDL1 levels, determined the immune responsiveness to PD1-based immune checkpoint therapy in OSCC patients [29]. The expression of the inhibitory receptor PD1 through lymph node and tumour-infiltrating regulatory T cells has been shown to be correlated with lymph node metastasis in pancreatic ductal adenocarcinoma [30]. The presence of metastatic neck nodes and tumour recurrence is associated with poor prognoses [31, 32]. A recent study showed that PD1 protein expression was significantly related to PDL1 expression, a higher tumour-infiltrating lymphocyte abundance, and distant metastasis [33]. Nonetheless, the relationship between lymph node metastasis of tumour cells and immune checkpoints remains unclear. Our study demonstrates that increasing the PD1 expression of CD4^+^ T cells in LN^+^ may promote lymph node metastasis, thereby suggesting that blocking PD1 may have therapeutic potential in these patients.

Activated immune and cancer cells often share metabolic similarities in the tumour microenvironment. A striking increase in glycolysis is the main feature of T-cell activation [34]. Glucose can be used by T cells to support effector functions [35]. Accordingly, T cells do not have extensive internal glycogen stores, making them highly dependent on the uptake of extracellular glucose to meet the increased metabolic needs during an immune response [10]. A study by Bengsch showed that PD1 regulates glycolysis and the mitochondrial function of virus-specific CD8^+^ T cells in chronic lymphocytic choriomeningitis virus infections [36]. Melanoma patients with high expression of glycolysis-related genes also showed worse progression-free survival rates following anti-PD1 treatment [37]. Our study has demonstrated that increasing PD1 in CD4^+^ T cells in LN^+^ is associated with glycolysis-related enzymes, thereby indicating that increased PD1 in CD4^+^ T cells inhibits antitumour immunity and is associated with glucose metabolism and aerobic glycolysis. Furthermore, PD1 and Hk2 of CD4^+^ T cells also increased in P-LN^+^ compared to N-LN^+^. This suggests that Hk2 may be a key enzyme in glycolysis, thereby contributing to the progression of metastatic lymph nodes in OSCC.

## Conclusions

In summary, our study suggests that lymph node metastasis and recurrence in OSCC are associated with increases in PD1 and glycolysis in CD4^+^ T cells; Hk2 may be a key enzyme in glycolysis contributing to the progression of metastatic lymph nodes in OSCC.

## Electronic supplementary material

Below is the link to the electronic supplementary material.

Supplementary Material 1

## Data Availability

The datasets used and/or analyzed during the current study are available from the corresponding author upon reasonable request.

## List of abbreviations

OSCC: Oral squamous cell carcinoma
LN+: Metastatic lymph node
FCM: Flow cytometry
