# Supplementary material for: Increased levels of PD1 and glycolysis in CD4+ T cells are positively associated with lymph node metastasis in OSCC
Source: BMC Oral Health. 2023 Jun 3;23:356. doi: 10.1186/s12903-023-03043-6 (PMC10239138; doi:10.1186/s12903-023-03043-6)
Supplement: Supplementary file 1 — Supplementary Material 1 [file 12903_2023_3043_MOESM1_ESM.docx]

Supplementary table 1. Sequence of qRT-PCR

| Primers | | Forward | Reverse | |  |
| --- | --- | --- | --- | --- | --- |
| Glut1  HK2  HK3  Gpi  Tpi1  ENO1  PKM  MCT4  LDHa  PD1  PDL1  CTLA4  β-actin | GGCCATCTTTTCTGTTGGGG  ACGAGTTACCGGCTGAAGAA  TGTGAGGTTGGGCTAGTTGT  AAGGAAATCGCCCAACCAAC  GGGGCTTTTACTGGGGAGAT  TCTTTGACTCTCGCGGGAAT  ATGGCTGACACATTCCTGGA  CAGCTGGGCCACAAGTATTC  CAACATGGCAGCCTTTTCCT  CAGGGTGACAGAGAGAAGGG  TACTGTCACGGTTCCCAAGG  TGCAAGGTGGAGCTCATGTA  CCTGGCACCCAGCACAAT | | | CGCAGTACACACCGATGATG  TCCTGCCATACACCCACAAT  CAGGCCTCCGATCATCTTCT  CCATTGGTAGAAGCGTCGTG  CCAATGCAGGCGATTACTCC  CAACAGCCTTTGAGACACCC  AGAAGTTCAGACGAGCCACA  GGCTCCTGTCCAGTCATACA  ACCCACCCATGACAGCTTAA  CCTGGCTCCTATTGTCCCTC  GGAGAGCTGGTCCTTCAACA  CACATTCTGGCTCTGTTGGG  GGGCCGGACTCGTCATACT | |
